# Evaluation of the Impact of the Tiny Earth Project on the Knowledge About Antibiotics of Pre-university Students in the Province of Valencia on Three Different School Years (2017–2020)

**DOI:** 10.3389/fmicb.2020.576315

**Published:** 2020-11-19

**Authors:** Jose I. Bueso-Bordils, Beatriz Suay-García, Carolina Galiana-Roselló, Elisa Marco-Crespo, María-Teresa Pérez-Gracia

**Affiliations:** ^1^ Departamento de Farmacia, Universidad Cardenal Herrera-CEU, Valencia, Spain; ^2^ ESI International Chair@CEU-UCH, Departamento de Matemáticas, Física y Ciencias Tecnológicas, Universidad Cardenal Herrera-CEU, Valencia, Spain; ^3^ Departamento de Comunicación e Información Periodística, Universidad Cardenal Herrera-CEU, Valencia, Spain

**Keywords:** Tiny Earth, resistances, antibiotics, rational use, global awareness, knowledge surveys, citizen science

## Abstract

According to the World Health Organization (WHO), antibacterial resistance is a serious problem worldwide. In Spain, knowledge about the use of antibiotics is scarce, being the third country with the highest consumption of antibiotics in the world and the first in Europe. This problem is due, partly, to the abusive use of these drugs in human medicine, livestock, and agriculture. The objective of this study was to evaluate the impact that the Tiny Earth project has had on the antibiotic knowledge in pre-university students. To do this, a survey was conducted before and after the Tiny Earth project in three different school years (2017–2020) to 322 pre-university students belonging to seven schools in the province of Valencia. The survey consisted of 12 multiple-choice questions with a single valid answer. We observed 67.6% success at the beginning and 81.2% at the end. These data indicate that they correctly answered an average of 1.64 more questions after completing the project. In view of the results, we can affirm that the Tiny Earth project has contributed to an improvement in scientific knowledge and awareness of the correct use of antibiotics and the emergence of resistances by pre-university students, which could also be transmitted to their social environment, thus improving awareness global on these issues.

## Introduction

Antibacterial resistance has become a serious health problem worldwide (Prigitano et al., 2018) and is now spreading faster in comparison to the development of new molecules. It should be noted that only 8 of the 33 new antibiotics in development belong to new families, a small number considering the large number of bacterial resistances we are facing (Lewis, 2017). In fact, according to WHO, bacterial resistances will be the leading cause of death in 2050 and the biggest challenge in the field of Biomedicine in the 21st century (World Health Organization, 2016).

In Spain, knowledge about the use of antibiotics is scarce, being the third country with the highest consumption of antibiotics in the world and the first one in Europe (Klein et al., 2018). This problem is due, in part, to the misuse of these drugs in human medicine, livestock, and agriculture (Phillips et al., 2004). This problem is even more serious if we bear in mind that three quarters of the antimicrobial agents used in livestock overlap with antimicrobials used in humans (O’Neill, 2015). This has led to the so-called “antibiotic crisis,” result of the shared irresponsibility of health professionals, politicians, and consumers of antibiotics themselves, though in very different degrees of responsibility (Cisneros Herreros and Peñalva Moreno, 2018).

In this context, it seems important to promote educational initiatives on the proper use and prescription of antimicrobial drugs (Scaioli et al., 2015). Following this line of action, Tiny Earth (2018) appears as an innovative project of citizen, participatory, educational, and social science based on a crowdsourcing strategy for the exploration of microbial biodiversity in soils in search of new antibiotic-producing microorganisms (Valderrama et al., 2018). Motivation is reinforced by the fact that there already exist compounds described as antibacterials produced by microorganisms isolated from soil, namely malacidins and teixobactin (Hover et al., 2018). In this way, it motivates pre-university students toward choosing a scientific degree while addressing a global health threat such as the resistance of bacteria to antibiotics (Handelsman et al., 2016; Pernaute and Jiménez, 2017).

A crowdsourcing strategy involves outsourcing tasks, rather than being performed by institution staff, overseeing a large group of volunteers or community through an open call. The idea of crowdsourcing is relatively recent, it shares the perspective defended by some academic sectors of free and open access to scientific knowledge (Open Access) and benefits from advances in communication technologies (internet and social networks). It has been postulated for use in the field of Public Health and other aspects related to Biomedicine (Brabham et al., 2014; Bentzien et al., 2015) and, applied to the educational environment, can provide a collaborative and practical dimension that is highly motivating for students (Caruso et al., 2016).

At the same time that it instructs and motivates, Tiny Earth alerts, reports and raises awareness to society and future generations. It invites pre-university students to participate in a real international project that addresses a very relevant health issue such as the lack of effective antibiotics to fight the infections caused by multi-resistant bacteria, which are already immune to virtually our entire therapeutic arsenal (Davies and Davies, 2010). Throughout the lab lessons, the Tiny Earth program focuses on the idea of discovery, in which students from around the world carry out creative fieldwork and laboratory research on soil samples in search of new antibiotic-producing microorganisms (Davis et al., 2017).

The implantation for the first time at the CEU Cardenal Herrera University (CEU UCH), sponsored by the Spanish Microbiology Society within the D+D group, of the successful educational and informative project of American origin Tiny Earth raises an important and original novelty compared to the United States project. It involves integrating two educational levels, pre-university and university, by implementing an ambitious strategy of service learning, which implies that the teaching activities and strategies used in the training of university students must have a direct impact on the community and society, integrating concepts of active learning, practical teaching, group work, and social volunteering. Indeed, the learning aspect of this project is not limited to the knowledge that pre-university students can acquire, since university students also acquire a series of new skills related to their knowledge transmission skills, teamwork, lab work, and expertise on the subject of antibacterial resistance.

To launch the Tiny Earth project, we recruited undergraduate students who had already taken courses in the field of Microbiology and students in the field of Communication Degrees in various degrees of the CEU UCH, to highlight the importance of divulgation and scientific communication and raise awareness about this health issue. This project was carried out in five private and state-funded schools in the province of Valencia during the first year (2017–2018). The project was expanded the second year (2018–2019) to include two more schools. In its third year (2019–2020), the schools remained the same.

The aim of this study is to evaluate the impact that the Tiny Earth project has had on the knowledge about antibiotics of the participating pre-university students. The results of our survey, which involved people of a very narrow age range, cannot be extended to the entire Spanish population. Nevertheless, they provide valid elements to promote initiatives aimed at a more conscious use of antibiotics.

## Materials and Methods

The Tiny Earth project consists of five practical sessions, challenging young students to discover novel bioactive-producing microorganisms from soil samples as well as raising awareness about antibiotic resistance and their appropriate use. Throughout 3 academic years, 19 Tiny Earth teams, each consisting of three to five undergraduate students or Tiny Earth Teaching Assistants (Tiny Earth TAs) led by one University teacher or Tiny Earth Partner Instructor (Tiny Earth PI).

**Figure 1:**
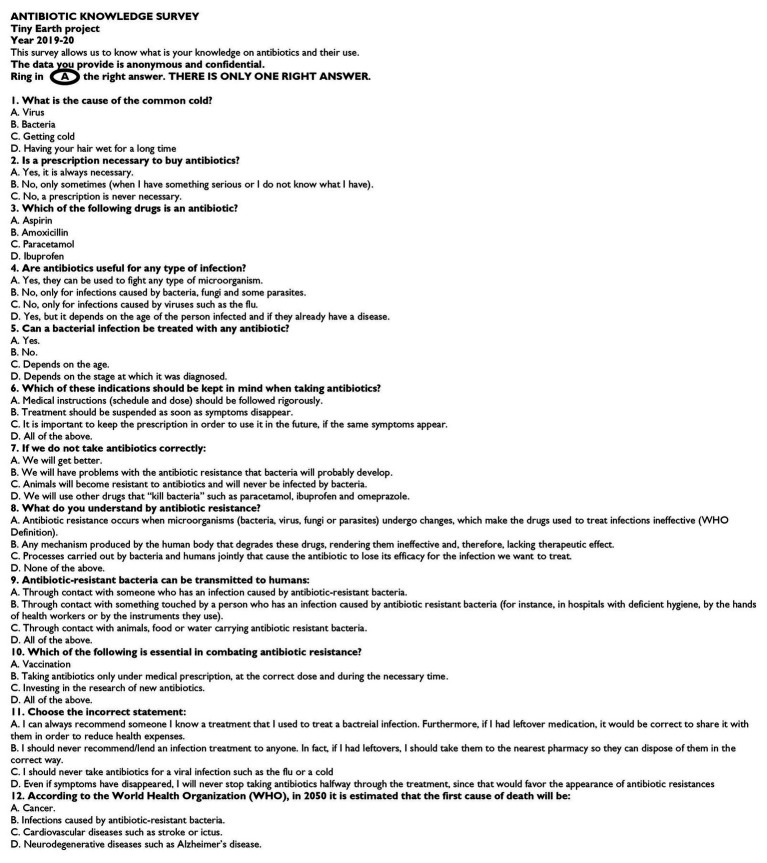
Tiny Earth project’s survey (in English) consisting of 12 test questions with a single valid answer.

To evaluate the pedagogical impact of the project, 322 pre-university students filled in a survey at the beginning (practical 1) and at the end (practical 5) of their participation in the Tiny Earth project, which consisted of 12 multiple-choice questions with one correct answer. We wrote this survey in Spanish and English (Figure 1) and we handed it out in one language or the other, depending on the school. The aim of this survey was to assess the level of knowledge about the appropriate use of antibiotics and the level of perception regarding the problem of antibiotic resistances. This survey was prepared based on questions asked by Tiny Earth TAs and Tiny Earth PIs who participated in the project.

Unlike other studies in which respondents were required to provide certain personal data for further demographic analysis (World Health Organization, 2015), in our case, we decided to carry out the surveys anonymously in order not to exert added pressure to students and preserve the relaxed albeit rigorous character of the activities carried out.

Statistical parameters (such as mean, standard deviation, and variances) were calculated using the Microsoft Excel 2016 program. The Fisher-Snedecor test (*F*-test) was used to analyze the equality of the variances and the Student’s *t*-test was used to determine statistical significances, both tests performed at 95% confidence interval (CI).

## Results

**Figure 2:**
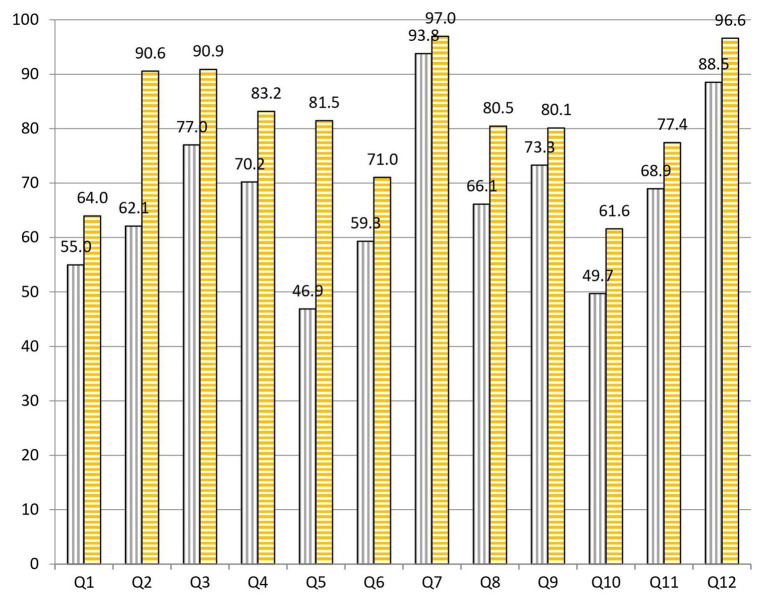
Percentages of correct answers to the 12 questions of the test carried out by pre-university students at the start (purple bars with vertical stripes) and at the end (yellow bars with horizontal stripes) of the Tiny Earth project during the courses 2017–2020.

After analyzing the survey results, we observed 67.6% (8.11/12) of success at the beginning and 81.2% (9.74/12) at the end of the project. These data indicate that students correctly answered an average of 1.64 more questions after completing the practical sessions of the project (Figure 2). The statistical data reveal that there are significative differences at 95% CI when comparing the overall results at the beginning and at the end of the project (Table 1).

**Table 1:** Statistical parameters at 95% confidence interval of the comparison between results obtained at the start and at the end of the project.

	Standard deviation	Variance	*F*-test	Variance equality	Weighted variance	*t*-test	Significative differences
Q1 start	5.048	16.988	5.855	Equal	87.345	1.131	No
Q1 end	12.215	99.472					
Q2 start	11.058	81.518	5.861	Equal	71.570	4.200	Yes
Q2 end	4.568	13.909					
Q3 start	10.005	66.735	15.951	Equal	53.189	2.378	No
Q3 end	2.505	4.184					
Q4 start	11.192	83.504	8.390	Equal	70.093	1.997	No
Q4 end	3.864	9.953					
Q5 start	37.219	923.491	144.997	Unequal	697.395	2.133	No
Q5 end	3.091	6.369					
Q6 start	5.698	21.645	18.052	Equal	309.290	0.783	No
Q6 end	24.210	390.741					
Q7 start	2.211	3.259	5.388	Equal	2.898	2.481	No
Q7 end	0.952	0.605					
Q8 start	4.018	10.765	2.120	Equal	25.188	3.512	Yes
Q8 end	5.850	22.818					
Q9 start	7.473	37.235	1.726	Equal	44.104	1.190	No
Q9 end	5.688	21.569					
Q10 start	13.975	130.197	2.380	Equal	138.681	1.265	No
Q10 end	9.059	54.711					
Q11 start	13.293	117.802	3.371	Equal	114.561	1.020	No
Q11 end	7.240	34.946					
Q12 start	5.389	19.358	15.928	Equal	15.430	2.327	No
Q12 end	1.350	1.215					
Overall start	3.818	9.717	9.165	Equal	8.083	5.998	Yes
Overall end	1.261	1.060					

**Figure 3:**
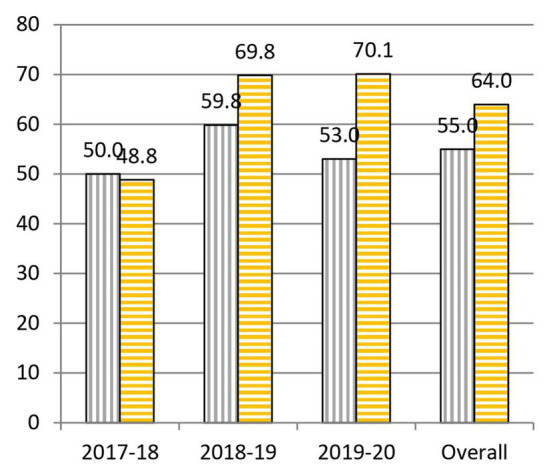
Percentages of correct answers to Question 1 “What is the cause of the common cold?” at the start (purple bars with vertical stripes) and at the end (yellow bars with horizontal stripes) of the Tiny Earth project during the courses 2017–2020.

**Figure 4:**
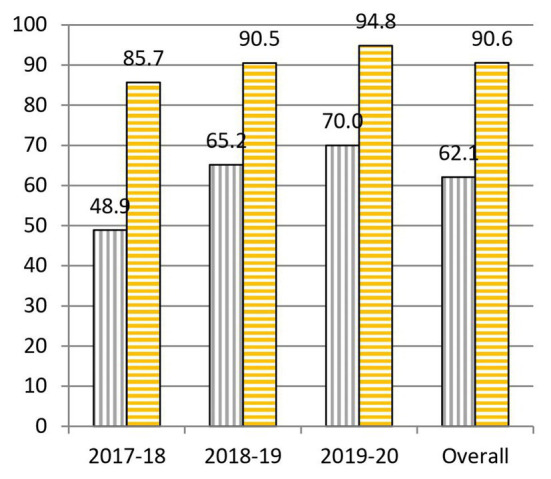
Percentages of correct answers to Question 2 “Is a prescription necessary to buy antibiotics?” at the start (purple bars with vertical stripes) and at the end (yellow bars with horizontal stripes) of the Tiny Earth project during the courses 2017–2020.

**Figure 5:**
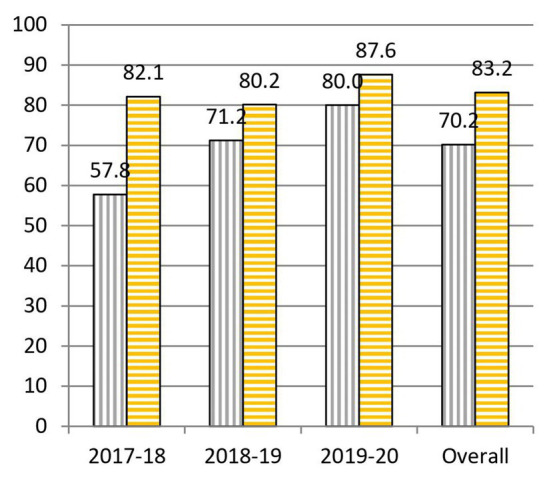
Percentages of correct answers to Question 4 “Are antibiotics useful for any type of infection?” at the start (purple bars with vertical stripes) and at the end (yellow bars with horizontal stripes) of the Tiny Earth project during the courses 2017–2020.

**Figure 6:**
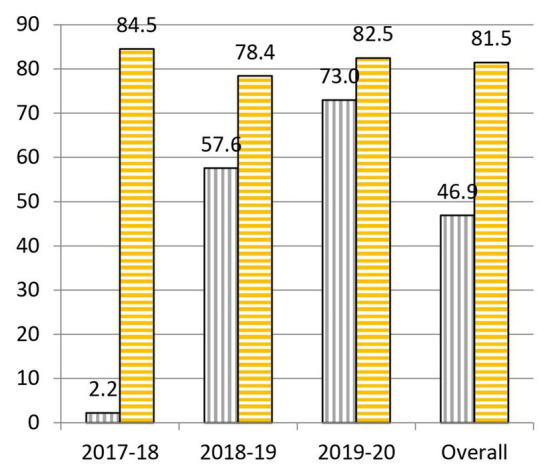
Percentages of correct answers to Question 5 “Can a bacterial infection be treated with any antibiotic?” at the start (purple bars with vertical stripes) and at the end (yellow bars with horizontal stripes) of the Tiny Earth project during the courses 2017–2020.

**Figure 7:**
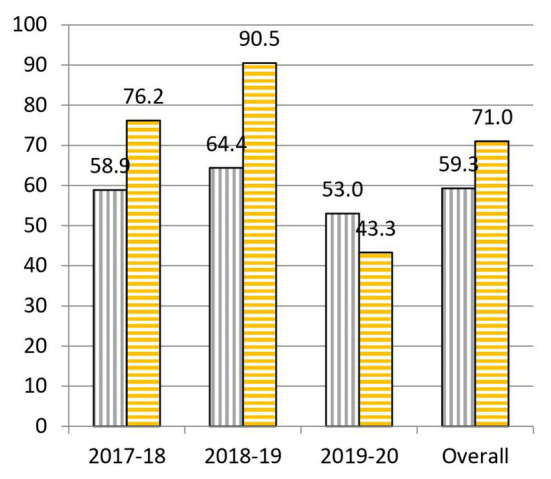
Percentages of correct answers to Question 6 “Which of these indications should be kept in mind when taking antibiotics?” at the start (purple bars with vertical stripes) and at the end (yellow bars with horizontal stripes) of the Tiny Earth project during the courses 2017–2020.

**Figure 8:**
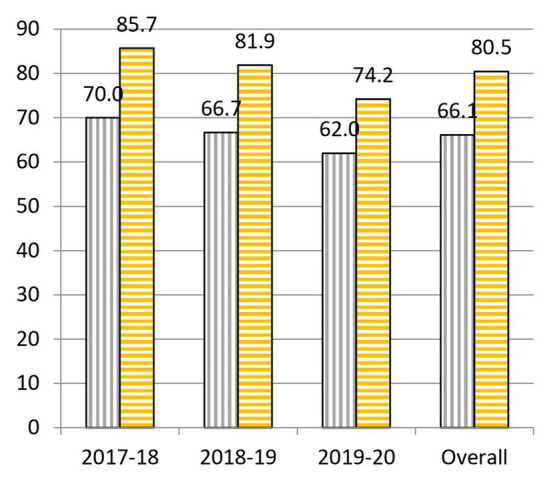
Percentages of correct answers to Question 8 “What do you understand by antibiotic resistance?” at the start (purple bars with vertical stripes) and at the end (yellow bars with horizontal stripes) of the Tiny Earth project during the courses 2017–2020.

When we analyze the results before and after the project, we highlight the following data: to question 1, “What is the cause of the common cold?,” 55.0% was correctly answered at the beginning and 64.0% at the end of the practical sessions (Figure 3). To question 2, “Is a prescription necessary to buy antibiotics?,” 62.1% was correctly answered at the beginning and 90.6% at the end of the practical sessions (Figure 4). To question 4, “Are antibiotics useful for any type of infection?,” 70.2% was correctly answered at the beginning and 83.2% at the end of the practical sessions (Figure 5). To question 5, “Can a bacterial infection be treated with any antibiotic?,” 46.9% was correctly answered at the beginning and 81.5% at the end of the practical sessions (Figure 6). To question 6, “Which of these indications should be kept in mind when taking antibiotics?,” 59.3% was correctly answered at the beginning and 71.0% at the end of the practical sessions (Figure 7). To question 8, “What do you understand by antibiotic resistance?,” 66.1% was correctly answered at the beginning and 80.5% at the end of the practical sessions (Figure 8). To question 10, “Which of the following is essential in combating antibiotic resistance?,” 49.7% was correctly answered at the beginning and 61.6% at the end of the practical sessions (Figure 2). The differences in the results before and after the project have proven to be statistically significant at 95% CI for questions 2 and 8 (Table 1).

## Discussion

From the results of the survey, in which there has been an increase close to 14% when comparing the number of questions answered correctly at beginning and at the end of the sessions, we can affirm that there has been a significant overall improvement in scientific knowledge from pre-university students participating in this project.

Nevertheless, we do believe that the results obtained in some of the questions asked deserve special attention, either because the difference in the correct answers to a specific question has been negligible, or because this difference has been especially noteworthy.

In question 1, “What is the cause of common cold?,” a slight drop (1.1%) was detected between the answers at the beginning (50.0%) and at the end (48.9%) of the project during the course 2017–2018 (Figure 3). A possible explanation for these data would be that, having carried out sessions dedicated mainly to bacteria, it might had influenced the perception that diseases are always caused by bacteria. We felt that the results on question 1 were too low on the first course, so in later editions, we tried to deepen our explanation regarding other diseases caused by other microorganisms, which gave place to a significant rise in the percentage of correct answers (10.0% in 2018–2019 and 17.1% in 2019–2020). We could say that the explanation was more efficient or effective in the following courses since, without changing it, it was more emphasized and better understood.

Question 2, “Is a prescription necessary to buy antibiotics?,” underwent a very considerable change with a 28.5% overall increase in correct answers given before and after the project (Figure 4). These data are probably due to the emphasis shown by Tiny Earth TAs and Tiny Earth PIs about the risks, both for the patient and for society, associated with the sale of antibiotics without a prescription (Guinovart et al., 2018). We would like to highlight that, year after year, the percentage of correct answers at the end has not ceased to increase (85.7% in 2017–2018, 90.5% in 2018–2019, and 94.8% in 2019–2020). Thus, we can conclude that the tendency of the gap between the percentage of correct answers before and after the project to shrink has more to do with the fact that students had a better knowledge on this question to begin with (48.9% of correct answers at the beginning of course 2017–2018, 65.2% in 2018–2019, and 70.0% in 2019–2020).

Another question that underwent a remarkable change, with a 13.0% increase in correct answers before and after the project was question 4, “Are antibiotics useful for any type of infection?” (Figure 5). Behind this improvement could be the continuous reminder by Tiny Earth TAs and Tiny Earth PIs throughout all practical sessions that antibiotics are indeed not useful for all infections. Similarly to question 2, even though the percentage of correct answers at the end of the project was quite high (82.1% in 2017–2018, 80.2% in 2018–2019, and 87.6% in 2019–2020), the general knowledge about this question before the project grew year after year (57.8% in 2017–2018, 71.2% in 2018–2019, and 80.0% in 2019–2020), reason why yearly gaps have decreased.

In question 5, “Can a bacterial infection be treated with any antibiotic?,” there has been a striking increase (34.6%) in the percentage of correct answers before and after the project (Figure 6). This could be due to the emphasis shown during the project, in which we explained that not all bacteria are alike, that they can cause various and very different infectious diseases, in addition to the own experience that pre-university students have had when viewing the different degrees of antibiosis against the same bacteria in the lab. A similar pattern to question 4 has been obtained. The percentage of correct answers at the end of the project were quite high (84.5% in 2017–2018, 78.4% in 2018–2019, and 82.5% in 2019–2020), the general knowledge about this question before the project grew year after year (2.2% in 2017–2018, 57.6% in 2018–2019, and 73.0% in 2019–2020), reason why yearly gaps have also decreased.

In question 6, “Which of these indications should be kept in mind when taking antibiotics?,” there has been an overall increase of 11.7% correctly answered questions between the beginning and the end of the practical sessions (Figure 7). However, if we focus on the yearly data, very good results were obtained in the first two courses (17.3% increase in 2017–2018 and 26.1% in 2018–2019), but there was a significant drop (9.7%) of correct answers between the beginning and the end of this course’s project. This result serves as a lesson to not to forget the importance of the rational use of antibiotics. Therefore, we should put a stronger emphasis on this matter in the coming years.

In question 8, “What do you understand by antibiotic resistance?,” there has been an overall increase of 14.3% in correct answers from the beginning to the end of the practical sessions (Figure 8). One of the reasons for these good results might be, besides the theoretical explanations on the concept of resistance given throughout the project, the fact that they have been able to recognize resistances visually by observation of inhibition halos.

In question 10, “Which of the following is essential in combating antibiotic resistance?,” there has been an overall increase of 11.9% in correct answers from the beginning to the end of the practical sessions (Figure 2). Even though there have been questions with less improvement between the beginning and the end, it is the question with the lowest success rate at the end of the project (61.6%). This might be due to the fact that it is a question in which all the answers are correct and, therefore, they must have learned all the concepts stated in the question.

We must say that our first goal was not to obtain uniform results from the tests. However, after analyzing the results on our first year, we tried to do some improvements in our presentations based on that information and, thus, results became more uniform for the last 2 years. However, we sincerely hope that teachers who may want to start a similar project could benefit from our findings.

We should also note that in schools where the number of students has been lower, both the results and collaboration have been more positive because the sessions were held more calmly and with a more personalized attention. In schools where there have been more students, although the results have also been satisfactory, attention could not have been so individualized. These results are consistent with various studies showing that while a significant proportion of the general public still believe that antibiotics are an effective treatment for cold symptoms, they also report increased awareness of antibiotic resistance (European Commission, 2013; Carter et al., 2016).

Chronic infectious diseases such as HIV and tuberculosis and emerging infections with the potential for rapid expansion such as the 2014 Ebola epidemic and the 2020 COVID-19 pandemic remain a substantial global health threat to mankind (Shahmanesh et al., 2020). Infectious diseases have profound effects beyond health, especially on local and global economies, which intensifies existing socioeconomic fragilities (United Nations Development Programme and International Federation of Red Cross and Red Crescent Societies, 2017). However, the prevalence of antimicrobial resistance grows by the year and we will soon be speaking about millions of deaths from antibiotic-resistant infections (Collignon et al., 2018).

We think that there is an urgent need for educational and awareness programs integrating methods to optimize the prevention of, and response to, infectious diseases. This might mean training science teachers and broadcasters to improve their dissemination on human health (Samet and Woodward, 2019).

## Conclusion

The global experience of the Tiny Earth project has been very positive. Participation in the Tiny Earth project has positively contributed to the development of scientific interest, the awareness on the proper use of antibiotics, and the emergence of resistances in pre-university studies, as well as improved their skills in the laboratory.

Likewise, in view of the results obtained with the survey, we can affirm that the students participating in this project have improved their scientific knowledge. Besides, these newly learned skills may also be transmitted to their social environment, thus improving the overall awareness of this issue. We can also conclude that the Tiny Earth project has awakened and expanded the scientific training and interest in students.

We would also like to highlight the need for new approaches in order to reach the general public in the field of infectious diseases.

## Data Availability Statement

The original contributions presented in the study are included in the article/supplementary material, and further inquiries can be directed to the corresponding author.

## Ethics Statement

Ethical approval was not provided for this study on human participants because no personal data was collected. We asked for informed consent given that the students that participated in the project where under 18 years old. Written informed consent to participate in this study was provided by the participants’ legal guardian/next of kin.

## Author Contributions

M-TP-G was responsible for the project design, conception and management and the integrity of the work, and overall supervision. JB-B, BS-G, CG-R, and M-TP-G performed the practicals. EM-C was responsible for the broadcasting of the project. JB-B wrote the first draft of the manuscript. BS-G, CG-R, and EM-C wrote sections of the manuscript. All authors contributed to interpretation of the data, manuscript revision, read, and approved the manuscript.

### Conflict of Interest

The authors declare that the research was conducted in the absence of any commercial or financial relationships that could be construed as a potential conflict of interest.
